# Association of ScV-LA Virus with Host Protein Metabolism Determined by Proteomics Analysis and Cognate RNA Sequencing

**DOI:** 10.3390/v14112345

**Published:** 2022-10-25

**Authors:** Juliana Lukša, Enrika Celitan, Elena Servienė, Saulius Serva

**Affiliations:** 1 Department of Biochemistry and Molecular Biology, Life Sciences Center, Vilnius University, LT-10257 Vilnius, Lithuania; 2 Laboratory of Genetics, Nature Research Centre, LT-08412 Vilnius, Lithuania

**Keywords:** *Saccharomyces cerevisiae*, yeast virus, *Totiviridae*, dsRNA, proteomics, transcriptomics

## Abstract

*Saccharomyces* yeasts are highly dispersed in the environment and microbiota of higher organisms. The yeast killing phenotype, encoded by the viral system, was discovered to be a significant property for host survival. Minor alterations in transcription patterns underpin the reciprocal relationship between LA and M viruses and their hosts, suggesting the fine-tuning of the transcriptional landscape. To uncover the principal targets of both viruses, we performed proteomics analysis of virus-enriched subsets of host proteins in virus type-specific manner. The essential pathways of protein metabolism–from biosynthesis and folding to degradation–were found substantially enriched in virus-linked subsets. The fractionation of viruses allowed separation of virus-linked host RNAs, investigated by high-content RNA sequencing. Ribosomal RNA was found to be inherently associated with LA-lus virus, along with other RNAs essential for ribosome biogenesis. This study provides a unique portrayal of yeast virions through the characterization of the associated proteome and cognate RNAs, and offers a background for understanding ScV-LA viral infection persistency.

## 1. Introduction

*Saccharomyces* yeasts are broadly scattered in higher organisms’ environment and microbiota, including humans [1,2]. Yeasts also are a profitable host as a protein factory [3]. A killing phenotype, a biocidal feature of yeast, was discovered to be a desired trait in industrial strains for the control of spoilage microorganisms and the preservation of the quality of food products and beverages [4]. The yeast killer property is frequently established by two persistent *Totiviridae* viruses, LA and M, together ensuring the synthesis of killer toxin in a host cell. The prevalence of killer M viruses among *S. cerevisiae* strains is considered limited [5], yet LA is common in a variety of wild, industrial, and laboratory yeasts [6].

The LA virus dsRNA genome encodes the coat protein Gag and the RNA-dependent RNA polymerase GagPol. To enable the development of a killer phenotype in the host cell, these two proteins assemble in capsids that provide replication and transcription of LA and also satellite M virus [7]. LA virus’ extracellular phase is unknown [8], and the presence of LA alone appears symptomless in yeast cells [9]. The amount of LA dsRNA was found comparable to that of cellular rRNA [10], contributing significantly to the total RNA content. The LA capsid is hijacked by the M virus for its maintenance, which solely codes for a preprotoxin targeting virus-free cells after maturation and secretion [7]. This reciprocal interaction of the LA and M viruses is functionally connected in precise synergy with the host cell.

For the host, the LA virus maintenance appears to be less impactful than that of satellite M, as only a few genes have previously been identified as being crucial [8]. The factors and mechanisms contributing to LA virus maintenance are less studied, however LA dsRNA tends to be more resilient to elimination than M dsRNA [11]. Recent research found that the overproduction of trimmed LA capsid protein completely eliminates the viral genome and is independent of LA type, implying comparable mechanisms of LA maintenance in non-killer yeasts [12].

LA virus genome contains a short 5′ untranslated region, followed by a single open reading frame (ORF) and a3′ untranslated region. Gag, the capsid protein, is encoded at the 5′proximal region of the genome, while 101 nt from its coding sequence end starts a robust secondary structure, region resulting in -1 frameshift of ribosome and production of fusion protein GagPol [11,12].

M dsRNA encodes killer toxin, which is produced in preprotoxin form, with a 3′ polyA segment of several hundred nucleotides and a following 3′ untranslated region, hosting encapsidation and replication signals [8]. The K1 toxin, by far the most studied, binds to yeast cell envelope glucans as does the K2 toxin [13,14,15]; both form pores in the cytoplasmic membrane of sensitive cells. K28 is endocytosed via Erd2p-coupled uptake and moves into the nucleus to block DNA synthesis [16], whereas Klus’ mode of action remains to be solved [17].

The replication of LA virus is regulated by the host cell. Since nascent LA mRNA lacks both a cap and polyA tail [13], its 5′ terminus is susceptible to degradation by exonuclease Xrn1/Ski1, whereas cytoplasmic exosome degrades mRNA without a polyA tail at the 3′ terminus [14,15]. Xrn1 regulates a variety of cellular processes, including meiosis [16], filamentous growth [18], RNA turnover [19], control of telomere length [20], respiration [21], and autophagy [22]. The exosome function is connected to the SKI complex, which requires the Ski2, Ski3, Ski7, and Ski8 proteins for exosome target identification [23].Disruption of one of these SKI genes allows markedly higher levels of viral dsRNA, and in the presence of M virus the production of killer toxin leading to so called “super-killer” phenotype is elevated [15]. The virus has developed protective strategies to circumvent the restriction measures. A cap-snatching mechanism for LA protein translation has been proposed [24]. Although triphosphatase and kinase activities are associated with LA virus-like particles [25,26], the ends of the LA positive strand are 5′-diphosphorylated by an yet unknown proteins. Diphosphorylation is required for viral transcription and functions as a viral RNA self-identity tag [27]. The copy number of LA dsRNA is negatively regulated by the mitochondrial porin Por1/2 and mitochondrial nuclease Nuc1 [28,29,30,31]. This regulation may be important for the survival of sporulating cells [30].

Several studies have observed the connection of LA virus amount to different metabolic conditions and sporulation [28,30,31]. Recently, host gene transcription patterns have been studied in yeast with different content of dsRNA viruses [27,32,33,34]. Transcriptome studies primarily target wild-type killer cells, therefore the effects of LA and M viruses overlap. Therefore, biological sensing of the LA virus maintenance in non-killer yeast remains a long-standing challenge in the field of yeast dsRNA viruses.

The replication and maintenance of LA virus are related to at least several host genes and metabolic conditions. LA replication has been reconstituted in vivo and is widely assumed to take place in the cytoplasm via a conservative transcription process [8,35]. N-terminal Gag acetylation by NatC N-acetyltransferase, encoded by the *MAK3*, *MAK10*, and *MAK31* genes, and other yet-unknown host factors are required for the assembly of LA virus particles [8,36,37,38]. These undetermined factors are most likely essential for host viability or substitute one another, thereby hindering their identification. The maintenance of yeast dsRNA viruses is highly sensitive to changes in ribosome structure; here, killer yeasts provide an excellent model for studying the function of ribosomal proteins [39].

Ribosomes play a significant role in LA propagation [40]. Mutations in *RPL3*, *RPD3*, *SIN3*, *SAP30*, *RPL41A*, and *RPL41B* elevate the efficacy of programmed − 1 ribosomal frameshifting and frequently result in a reduction or loss of viral dsRNA(s), mostly impacting satellite M [29,39,41]. Cells with defective *RPL4A*, *RPL4B*, *MAK11*, and *MAK16* genes, which are involved in the production of 60S ribosomal subunits, have decreased LA dsRNA copy number and Gag protein levels [40]. It was proposed that 60S ribosomal subunits are required for the sufficient translation of LA capsid proteins; nevertheless, there were also exceptions, which were likely due to the differences in the LA mRNA sequences between LA variants [40]. During the adaptation to cell growth on glycerol, non-killer yeast mutants lacking mitochondrial pore protein generate enormous numbers of virus-like particles and LA dsRNA [31]. Ethanol-grown yeast cells exhibit higher levels of viral dsRNA than glucose-grown cells [31], which may be related to the finding that glucose represses *MAK10* [42]. The LA virus and mitochondrial DNA may also compete for Mak10 protein [42]. Double-stranded RNA viruses have been demonstrated to cover distinct chromosomal mutations and, in some instances, to be affected by a mitochondrial genome variant, suggesting that non-chromosomal components can interact to alter the host phenotype [43].

The main uses of proteomics in virus studies focus on the determination of the protein composition of virions, the structure and protein interactions of viral proteins, and the effects of viral proteins along with viral infection on the cellular proteome [44]. Some viruses are studied by multiple proteomics approaches, such as herpes simplex (HSV-1) [45], human immunodeficiency virus (HIV-1) [46,47], baculovirus [48], *flaviviriadae* family members, such as Zika virus [49,50]. An unbiased and direct registration of the interactions between the viral proteins and the host cellular proteins leading to the construction of virus-host cell interactome, such as tombusvirus [51] remains the significant use of proteomics approaches. Unfortunately, there have been few proteomics studies on particular contacts between the host proteins and the viral capsid proteins. For this type of analysis, a co-immunoprecipitation combined with liquid chromatography mass spectrometry (LC-MS) approach was used for porcine circovirus type 2 [52]. For analyzing changes in the host cell proteome over a time course of infection, the quantitation strategies such as stable isotope labeling by amino acids in cell culture (SILAC), isobaric tags for relative and absolute quantitation (iTRAQ), or tandem mass tag (TMT) are involved [53]. The amount of proteins measured across different cell conditions is significantly increased when isobaric labeling techniques are used. In particular, TMT approach has recently developed into a potent tool for proteome profiling [54] and was applied in numerous viral-host interaction investigations [55,56,57,58,59,60,61]. Proteomics studies have thus offered a direct insight into the pathophysiology involved in viral infection and demonstrated the dynamic interactions between the virus and the host.

Gene expression studies of *Totiviridae* virus impact on host demonstrated a moderate intensity, however broad transcriptional response to viral dsRNA elimination, supporting the notion of long-lasting co-adaptation [35,36]. The importance of the host background was revealed by different killer phenotypes of distinct strains bearing dsRNA viruses of the same type, also explained by a virus and host co-evolution in different populations [60]. LA virus seems to be less challenging to the host than the satellite M, as only a few host genes have been identified as critical for LA maintenance [27,33,34]. Furthermore, LA tends to be more resistant to elimination than M, suggesting a closer relationship with the host [61].

Our investigation focused on three isogenic *Saccharomyces cerevisiae* M437 strains with varying content of dsRNA viruses LA-lus and M2. In order to identify the virus-associated host factors, viral particles were separated with respect to viral dsRNA content by equilibrium centrifugation and quantitative proteomics analysis of selected fractions was performed. As the follow-up investigation, the virus-linked cellular RNAs have been addressed by sequencing analysis. In general agreement with transcriptomics data, a moderate response of the cell to the viral content has been observed. The major pathways of protein metabolism were found substantially enriched in virus-linked subsets: ribosome biogenesis, folding and proteasomal degradation. Ribosomal RNAs were found inherently associated with LA-lus virus, along with other RNAs essential for ribosome biogenesis. These findings substantiate the tight integration of virus with the essential pathways of host cell, therefore offering background for understanding ScV-LA viral infection persistency.

## 2. Materials and Methods

### 2.1. Yeast Strains and Growth Media

The *Saccharomyces cerevisiae* strain M437 (*wt* HM/HM [kil-K2]), carrying LA-lus and M2 dsRNA viruses, and those cured of either M2 dsRNA virus M437[L+M-] or both LA-lus and M2 viruses M437[L-M-] isogenic strains [33] were used for experiments. Yeast cultures were propagated in YPD medium (1% yeast extract, 2% peptone, 2% glucose).

### 2.2. Sample Preparation for Proteomics Analysis and RNA Sequencing

**Figure 1:**
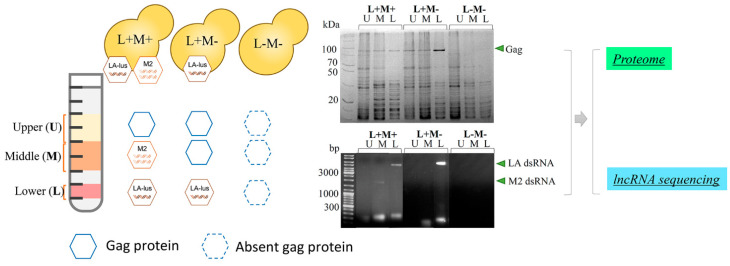
Workflow for characterization of virus-related changes in proteome and transcriptome of different fractions. (U)–upper fraction without viral dsRNA, (M)–M2 viral dsRNA bearing fraction, (L)–LA ds RNA bearing fraction.

Batch yeast cells were grown at 30 °C to mid-log phase (O.D.600 ~0.5) in 600 mL of YPD medium. Three yeast strains were harvested in biological triplicates. The supernatant was collected, centrifuged at 5000× *g* (5 min, 4 °C) and washed using distilled water. For further analysis, 2.3 g of cells per sample were used. The cell wall was removed with zymolyase as described in [62]. Spheroplasts were resuspended in 1 volume of AB buffer (20 mM Tris-HCL pH 7.5, 50 mM KCl, 10 mM MgCl_2_) with 1% PMSF. Yeast cell lysate was produced using glass bead disruption by 1 min blasts of vigorous vortexing, interspersed with 1 min rests of the sample on ice for 6 cycles. Glass beads were removed by centrifugation (1000× *g*, 10 min, 4 °C) following a second centrifugation to remove crude cell debris (10,000× *g*, 10 min, 4 °C). 0.25 volume of 25% PEG4000 in AB buffer with 2.5 M NaCl was added to supernatant and samples were incubated for 30 min on ice. After centrifugation for 10 min at 15,000× *g*, 4 °C the pellets were washed with 0.6 volume 5% PEG4000 in 1× AB, 3 M NaCl solution. After a short centrifugation, 220 µL of 1× AB buffer were added to dissolve the sediment. Finally, the samples were further purified by cesium chloride density gradient (1.37 g/cm^3^) ultracentrifugation at 127,000× *g* for 24 h. Fractions of 300 µL were collected and tested via total RNA extraction and electrophoresis, as well as SDS-PAGE. In total, nine fractions with LA-lus (L, lower fraction of high-density), M2 (M, middle) dsRNA and Gag protein, as well as capsid protein-only bearing fractions (U, upper fraction of low density) were used for proteomics analysis, as well as for RNA sequence analysis (Figure 1). Same steps were repeated with the strain M437[L+M-] and corresponding control fractions of strain M437[L-M-], all in triplicates, adding to a total of 27 samples.

### 2.3. RNA Extraction

50 µL of sample fraction were mixed with 800 µL TES buffer (0.1 M NaCl, 0.01 M Tris-Cl pH 7.5, 0.01 M EDTA, 0.2 percent SDS), then 600 µL of acidic (pH 5.5) phenol added before incubating for 30 min with moderate shaking. The upper aqueous phase was separated by centrifugation at 20,000× *g* for 20 min. and treated with 60 µL 3 M NaAc pH 5.2 and 660 µL 2-propanol.The pellet was recovered by centrifugation at 15,000× *g* for 10 min, then washed with cold 75% ethanol and resuspended in 20 µL of nuclease-free water.

### 2.4. Proteomics Analysis

All samples were concentrated 4x on the Microcon YM-100 column (Millipore, Bedford, MA, USA). An additional desalting step was performed with ZebaTM Spin 7k MWCO columns (Thermo Fisher Scientific, Waltham, MA, USA) equilibrated with AB buffer. Further sample preparation and LC-MS/MS analysis was performed at Proteomics Core Facility of EMBL (Heidelberg, Germany), using a 10-plex TMT labeling approach to enable simultaneous quantification of replicates and reference sample. Only proteins that were quantified with two unique peptide matches were included for analysis. Moreover, only proteins that were quantified in at least 2/3 of the replicates were kept for analysis. The significance of the changes in protein abundance, as well as their log2 fold changes (log2FC), across the three conditions was determined using the Student’s *t* test. We used a *p* 0.05 criterion and an absolute log2FC > 0.5 to categorize the changes in abundance.

### 2.5. RNA Extraction for Sequence Analysis

Cognate RNA from sample fractions was isolated using TRIzol reagent (Invitrogen, Carlsbad, CA, USA), following the manufacturer‘s guidelines. In each sample fraction, 1:1 v/v TRIzolTM reagent was added and incubated for 5 min at room temperature before adding 1:1:1 v/v chloroform (Sigma-Aldrich, St. Louis, MO, USA) and incubating for 5 min at room temperature with continuous shaking. The samples were centrifuged for 20 min, 18,000× *g* at 4 °C. Aqueous phase was transferred to a new 1.5 mL microtube, RNA precipitated by an equal volume of isopropanol (Sigma-Aldrich, St. Louis, MO, USA) at 4 °C for 15 min. Samples were centrifuged for 10 min, 18,000× *g* at 4 °C. The supernatant was discarded, the pellet was washed with 140 µL of 70% ethanol (Sigma-Aldrich, St. Louis, MO, USA). The ethanol was removed after 18,000× *g* 1 min centrifugation. The pellet was dried at room temperature and the RNA was solubilized in 60 µL of nuclease-free water (Thermo Fisher Scientific, Vilnius, Lithuania). RNA quality and quantity were assessed by agarose gel electrophoresis and a Nanodrop 2000 spectrophotometer (Thermo Fisher Scientific, Wilmington, DE, USA).

### 2.6. Processing of Cognate RNA Sequencing Data

Standard sample preparation for NGS (cDNA library preparation, quality checks) and sequencing were performed by Novogene Inc. using a paired-end 150 bp workflow on the Illumina NextSeq 550. CLC Genomics Workbench software, version 22.0.2, was used to investigate the raw sequencing reads (Qiagen, Aarhus, Denmark). During import, the Illumina files (forward and reverse reads) were merged (quality scores: NCBI/Sanger or Illumina Pipeline 1.8). Trimming was performed on the reads with a quality score cutoff of 0.01 and a maximum limit of 1 unclear nucleotide. Trimmed adapter sequences were as follows:

5′ Adapter
5′-AGATCGGAAGAGCGTCGTGTAGGGAAAGAGTGTAGATCTCGGTGGTCGCCGTATCATT-3′
3′ Adapter
5′-GATCGGAAGAGCACACGTCTGAACTCCAGTCACGGATGACTATCTCGTATGCCGTCTTCTGCTTG-3′

Sequences shorter than 50 nucleotides were discarded. Trimmed readings were mapped to *S. cerevisiae* transcripts (GCF_000146045.2_R64_rna). Strict mapping requirements were implemented (mismatch, insertion and deletion costs: 2: 3: 3, respectively). All resulting RNA-Seq raw reads will be providedupon request.

### 2.7. Functional Annotation of Transcriptome and Proteome Data

Transcriptomics and proteomics data annotation was performed with YeastMine https://yeastmine.yeastgenome.org/yeastmine/begin.do (accessed on 4 April 2022). Volcano plots were obtained using VolcaNoseR available online: https://huygens.science.uva.nl/VolcaNoseR2/ (accessed on 12 April 2022). ShinyGO 0.76 available online: http://bioinformatics.sdstate.edu/go/ (accessed on 15 April 2022) was used to perform term enrichment analysis and retrieve metabolic pathways, only hits with *p*-value < 0.05 were considered. Using Venn web tool, Venn diagrams depicting the overlap of differentially expressed proteins (DEPs) in distinct categories were constructed online: http://bioinformatics.psb.ugent.be/webtools/Venn/ (accessed on 17 August 2022). Online YEASTRACT database provided a regulatory associations between transcription factors in *S. cerevisiae* http://www.yeastract.com/formfindregulators.php (accessed on 17 August 2022). The PPI network predicting physical and functional relationships between DEPs was created by STRING tool https://string-db.org/ (accessed on 24 August 2022 using high confidence score (0.8), visualized in cytoscape (v3.9.1) [63] and the molecular complex detection (MCODE) clustering algorithm was used to further analyze the data in order to find the subnetworks [64].

## 3. Results

### 3.1. Purification of Virus-Linked Sub-Proteomes

For the discovery of virus-associated host proteins, three isogenic yeast strains were employed: *Saccharomyces cerevisiae* M437[L+M+] bearing a complete killer system (LA-lus and M2 virus) (*wt* strain), M2 depleted M437[L+M-], and virus-free (M437[L-M-] as a reference [33]. Yeast cells were propagated in biological triplicates, collected after reaching the exponential growth phase and processed for virus fraction separation via isopycnic centrifugation on a gradient prepared in CsCl solution. The collected fractions were analyzed for the presence of viral capsid protein Gag by SDS-PAGE profiling and viral genomic dsRNA distribution (Figure 1). Fractions with large LA-lus (L, lower fraction of high-density), M2 (M, middle) dsRNA and Gag protein as well as capsid protein only bearing fractions (U, upper fraction of low density) were used for proteomics as well as RNA sequence analysis. In total, we prepared a set of nine samples, three biological replicates each. This approach enables to focus on proteins uniquely associated with both the different virus status and the identity of targeted virus genome dsRNA.

### 3.2. Proteomics Characterization of Virus-Associated Proteins

To address the protein composition of virus-bearing and virus-free yeast cell fractions, we performed proteomics analysis of each selected fraction by TMT labeling with following LC-MS/MS. The analysis of 9 samples and their replicates led to identification of 730 quantifiable proteins. The Principal Component Analysis (PCA) displays three largest sources of variation within the data set, showing that the samples present low variance within fractions independently of the strain (Figure S1). The protein ratio was obtained from each corresponding control sample fraction M437[L-M-] vs. M437[L+M+], or M437[L-M-] vs M437[L+M-]. The Student’s *t* test was used to determine the significance of changes in protein abundance as well as their log2 fold changes (log2FC) between the strain conditions (Table S1). To classify the abundance changes, we used a *p* < 0.05 criterion and an absolute log2FC > 0.5 (for further analyses). This resulted in a total of 102 and 92 unique upregulated proteins in all fractions, and 81 and 95 downregulated proteins when comparing two and one virus-bearing yeast strains, respectively (Figure S2). By applying the defined log2FC threshold regardless of yeast strain, 46 and 38 proteins were found to be significantly upregulated, and 46 and 52 proteins were downregulated in comparison to the control strain. Only 21% of proteins in M437[L+M+] and 14% in M437[L+M-] cells were up-regulated more than 1 log2FC, while down-regulation for −1 log2FC and lower was found for 8% of proteins equally in M437[L+M+] and M437[L+M-] cells in comparison to the reference virus-naïve strain M437[L-M-].

#### 3.2.1. Proteomes of M437[L+M+] L, M, U Fractions

To better understand the LA-lus and M2 virus-associated host proteins, we compared the changes in protein abundance between the corresponding *S. cerevisiae* M437[L+M+] and M437[L-M-] fractions. The presence of LA-lus virus in L fraction leads to altered expression levels of 52 proteins (>0.5 log2FC). The highest ratio between two cell types, more than two-fold, displays RAS-related Rsr1 protein (2.3 log2FC), also among the most up-regulated there are ribosomal 40S–60S proteins (Rpl13B, Rps12, Rpp2B, Rpp0, Rpl30, Rps28B, Rps22A of >1.4 log2FC), viral capsid GagPol protein (1.6 log2FC), proteasomal proteins (Pre1, Pre6, Scl1, Pup2, Pre9) and DNA-directed RNA polymerase I subunit Rpa190 (>1 log2FC). The most down-regulated proteins (higher than −1 log2FC) found in L fraction are chaperone proteins Hsp82, Ssa1 and Ssa2, tricalbin-3 Tcb3, manganese-transporting ATPase Spf1, phosphoinositide phosphatase Sac1, and Acyl-CoA desaturase1 Ole1.

M fraction harboring M2 virus contains 29 proteins of altered abundance. Only 3 proteins were upregulated more than 1 log2FC, these being the ribosomal proteins Rpl8A and Rpa8A, and the viral capsid GagPol protein. Among the 15 of down-regulated proteins Lat1, the component of pyruvate dehydrogenase complex, was the most down-regulated (−1.3 log2FC). The proteasome structural proteins (Pre5, Pre10, Pre7, Scl1) are present as well as in L fraction, but in this sample they are slightly down-regulated (less than 1 log2FC).

U fraction of M437[L+M+] cells, containing viral protein without dsRNA, has a few unique regulated proteins with only 12 up-regulated and 7 down-regulated proteins. Among up-regulated proteins we found proteasomal proteins of alpha subunit (Pre8, Pup2, Scl1), carboxypeptidase Prc1, 60S ribosomal proteins (Rpl20A, Rpl20B, Rpp2B) (<0.7–>0.5 log2FC). The most down-regulated protein in his group is triosephosphate isomerase Tpi1 (−0.92 log2FC).

#### 3.2.2. Proteomes of M437[L+M-] L, M, U Fractions

From a pairwise comparison of the M437[L+M-] and M437[L-M-] cells, we identified significantly regulated proteins in fractions of cells with higher number of viral particles than in M437[L+M+] cells. When considering the group of significantly regulated proteins in L fraction, we detected 19 up-regulated versus 22 down-regulated in this group. Among the most upregulated proteins (>1 log2FC), we found ribosomal 40S-60S proteins (Rps22A, Rpp2B, Rpp0, Rps17A, Rps0A), RAS-related Rsr1, proteasomal protein Pre1, Cdc39–the component of multisubunit protein complex found in all eukaryotes that helps to control RNA metabolism at all stages, and methylenetetrahydrofolate reductase Met12. The larger group of down-regulated proteins (>−1 log2FC) include YJL213W- protein of unknown function, which may interact with ribosomes as it is the most down-regulated, calcium/calmodulin-dependent protein kinase II Cmk2, exocyst complex component Exo84, S-adenosylmethionine permease Sam3, cell morphogenesis protein Tao3, GTP-binding protein Rho1, alpha-arrestin Ecm21, and phosphoinositide phosphatase Sac1.

The group of M fraction proteins includes 12 up-regulated and 23 down-regulated proteins. Mannosyltransferase Pmt1 is the most up-regulated protein (>1 log2FC). While the group of down-regulated proteins is >−1 log2FC, it consists mostly of ribosomal proteins (Rps22A, Rps1B, Rps0A, Rps7B, Rps17A, Rps16A, Rps2, Rpl1A) and protoplast secreted protein2 Psa2.

Finally, U fraction possess 12 up-regulated proteins, of which only virus structural proteins were up-regulated more than >1 log2FC. Other proteins of notable interest include proteasome 26S subunit Rpn2, structural maintenance of chromosomes protein1 Smc1, H/ACA ribonucleoprotein complex subunit 4 Cbf5, Mkt1 protein similar to nucleases, involved in propagation of M2 dsRNA satellite of LA virus, heat shock proteins (Ssa1, Hsp104), NADPH--cytochrome P450 reductase Ncp1, uncharacterized peptidase YFR006W, ribonucleoside-diphosphate reductase Rnr2, and 5′-3′ exoribonuclease1 Xrn1. 9 down-regulated proteins do not exceed −0.7 log2FC, among which are copper transport proteins (Ctr1, Ctr3), activator of SKN7 protein10 Ask10, ADP-ribosylation factor1 Arf1, enolase1 Eno1, pyruvate decarboxylase isozyme1 Pdc1, beta proteasome subunit Pre1, flavoprotein-like protein Ycp4, and glucose transporter Hxt2.

### 3.3. Functional Protein Analysis

**Figure 2:**
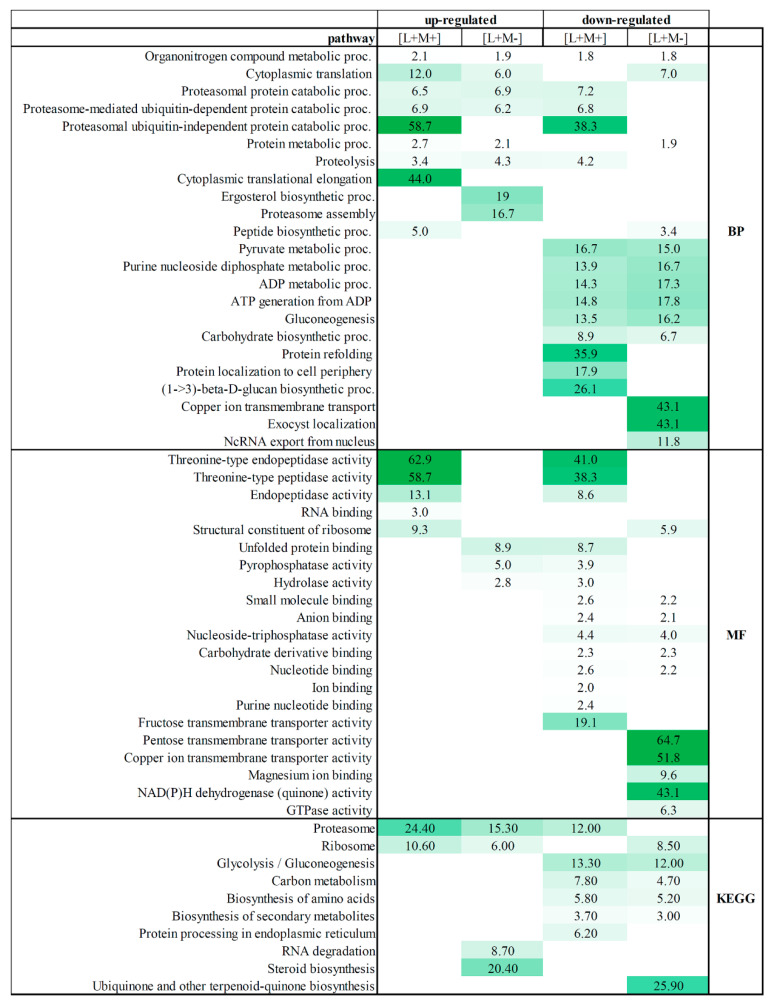
Gene ontology (GO) and KEGG enrichment analysis of statistically significantly up- and down-regulated DEPs, including biological process (BP), molecular function (MF). The number and color intensity represents fold enrichment.

To determine the overall cellular activities associated with viral presence or absence in all tested fractions, we examined the enrichment of the “biological process”, “function”, and “KEGG” gene ontology (GO) categories associated with proteins detected in all fractions (Fold enrichment (F.E.) values are presented in Table S2 and Figure 2).

Separate enrichment analysis of pathways for up- and down-regulated DEPs was performed. GO annotation analysis revealed that fractions from M437[L+M+] and M437[L+M-] strains share biological processes between up- and down-regulated proteins. Organonitrogen compound metabolic process is enriched in both strains and is both up- and down-regulated. Cytoplasmic translation process is shared by up-regulated proteins in both strains, and also is found in M437[L+M-] down-regulated protein fractions. Proteasomal-related protein catabolic processes, proteolysis are up-regulated in both strains, but some proteins of this process are also down-regulated in M437[L+M+] strain fractions. Uniquely up-regulated processes of M437[L+M+] fractions feature enriched cytoplasmic translation elongation, peptide biosynthesis, and proteasomal ubiquitin-independent protein catabolic processes. The ergosterol biosynthetic and proteasome assembly processes are clearly represented in the M437[L+M-] strain fractions. The functional annotation of down-regulated proteins fall in pyruvate metabolic process, purine nucleoside diphosphate, ADP metabolic processes, ATP generation from ADP, gluconeogenesis, and carbohydrate biosynthetic process in both strains. The group of proteins down-regulated in the M437[L+M+] strain is significantly enriched in GO terms including protein refolding, protein localization to cell periphery, and notably (1–>3)-β-D-glucan metabolism. Among the 95 downregulated proteins, only in LA virus-bearing strain copper ion transmembrane transport, exocyst localization, and ncRNA export from nucleus processes are enriched.

Proteins enriched in the activity of threonine-type endopeptidase/peptidase were detected in M437[L+M+] strain protein fractions as up-regulated, and part of proteins of that group were down-regulated. Only M437[L+M+] strain fractions observe proteins with RNA binding molecular function. Proteins that contribute to the structural integrity of a ribosome were up-regulated and enriched in M437[L+M+] strain fractions, whereas they are down-regulated in M437[L+M-] strain fractions. Unfolded protein binding, pyrophosphatase and hydrolase activity are enriched in down-regulated two virus-harboring strain fractions but not in M437[L+M-] strain fractions. The functional allocation of down-regulated proteins falls into small molecule, anion, carbohydrate derivative and nucleotide binding, along with nucleoside-triphosphatase activity in both strains. Proteins enriched in ion, purine nucleotide binding, and fructose transmembrane transporter activity are found in down-regulated M437[L+M+] protein fractions. Whereas down-regulated proteins in the M437[L+M-] fractions have the most significantly enriched GO terms linked to pentose and copper transmembrane transporter, magnesium ion bonding, NAD(P)H dehydrogenase, and GTPase activity enrichment.

The KEGG pathway analysis further demonstrates the enrichment of upregulated proteins in proteasomes and ribosomes in both strains. Part of the proteins are enriched in proteasomes but are down-regulated in two virus-bearing strains, while part of ribosome proteins is down-regulated in LA virus containing strain. In addition, both virus-infected strains have down-regulated biosynthesis of amino acids and secondary metabolites, carbon metabolism as well as glycolysis/gluconeogenesis.

In M437[L+M+] strain, only up-regulated proteins are enriched in ribosome biogenesis, RNA degradation and steroid biosynthesis KEGG pathways. Ubiquinone/other terpenoid-quinone biosynthesis is enriched and down-regulated only in M437[L+M-].

### 3.4. Transcription Factor Regulating

**Figure 3:**
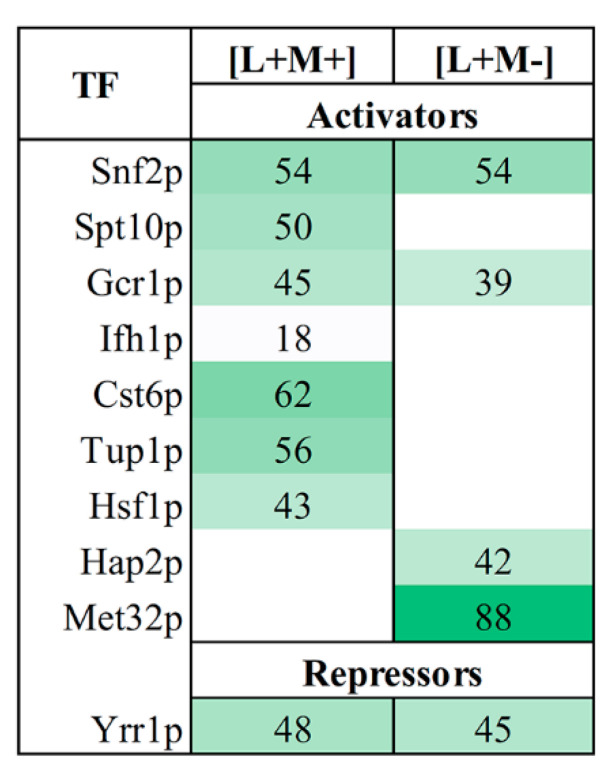
Reported regulators of DEPs in *S. cerevisiae*. Using the tools provided in the YEASTRACT database, upregulated genes were only searched for transcriptional activators, whereas downregulated genes were only searched for transcriptional repressors. The regulatory relationships are shown with the greatest degree of confidence supported by binding and expression evidence. The green color intensity represents the percentage of controlled genes.

We further used YEASTRACT analysis tool to investigate the transcription factors (TFs), which regulate gene transcription of proteins identified in our samples. We examined the reported transcription activators of up-regulated genes and inhibitors of down-regulated genes of proteins in all fractions (Figure 3). Our analysis revealed that 10 transcription factors regulate the expression of DEPs at a confidence with less than 0.05 *p*-value. Nine transcription factors are connected to the activation of up-regulated proteins, whereas one TF is linked to the repression of down-regulated proteins. All TFs are involved in regulation of transcription by RNA polymerase II. In both strains, three TFs (Snf2, Gcr1 and Yrr1) are engaged in the up- and down-regulation of DEPs. The expression of 62% of up-regulated proteins in M437[L+M+] can be induced by Cst6, whereas Yrr1-mediated repression is responsible for the majority of the down-regulated genes (48%) (Figure 3). The majority of positively regulated proteins in M437[L+M-] cells are connected to the action of the Met32 transcription factor (88%). Yrr1 has the ability to suppress the expression of the majority (45%) of down-regulated genes.

### 3.5. Identification of Significant Changes in Protein Abundance

**Figure 4:**
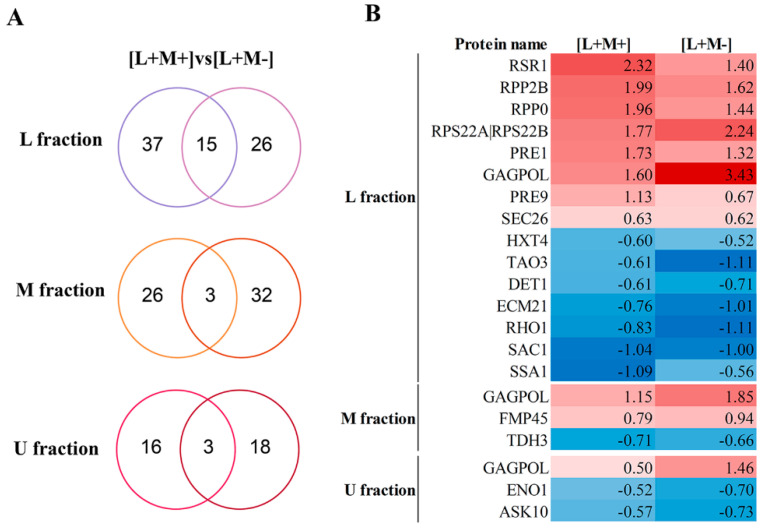
Analysis of DEPs in response to virus presence in distinct fractions using a Venn diagram and a heatmap. (**A**) Venn diagram showing overlap between fractions of M437[L+M+] vs. control and M437[L+M-] vs control DEPs. (**B**) Heatmap representing fold change of overlapped DEPs.

We discovered an enhanced up-regulation of virion capsid fusion protein GagPol in all fractions of both strains carrying virus, particularly in M437[L+M-]. When considering the groups of significantly regulated proteins in the lower (L) fractions that contain the LA virus, we detected 15 significantly regulated mutual proteins in M437[L+M+] and M437[L+M-] strains (Figure 4).

Of the 8 proteins with up-regulated change in abundance, there are structural components of ribosome (Rpp2B, Rpp0, Rps22A|Rps22B) and proteasome (Pre1, Pre9), Ras-type GTPase Rsr1, subunit of coatomer protein complex Sec26 and a GagPol protein. In the M437[L+M-] strain sample, we found that the GagPol and Rps22A|Rps22B component of the small (40S) ribosomal subunit are up-regulated higher by 1.83 and 0.47 log2FC, respectively, compared to M437[L+M-] strain. The group of 7 down-regulated proteins contains low-affinity glucose transporter Hxt4, cell morphogenesis protein Tao3, broad-range acid phosphatase Det1, alpha-arrestin Ecm21, GTP-binding protein Rho1, phosphoinositide phosphatase Sac1, heat shock protein Ssa1. In the middle fraction containing M2 virus in M437[L+M+] strain and in the corresponding fraction of M437[L+M-] strain without M2 virus, 3 proteins are shared and significantly regulated: upregulated GagPol and mitochondrial protein Rmp45, and downregulated glyceraldehyde-3-phosphate dehydrogenase Tdh3. In fraction M, the difference in GagPol between strains is lowest of 0.6 log2FC. The upper fraction without viral dsRNA in both strains increased the GagPol amount and downregulated a phosphopyruvate hydratase Eno1 and regulator of the Fps1 glycerol channel Ask10.

### 3.6. STRINGAnalysis

**Figure 5:**
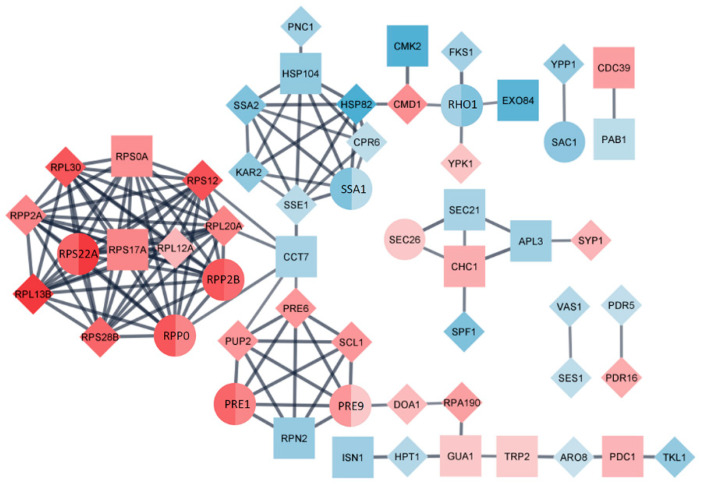
Protein-protein interaction (PPI) network of identified DEPs in L fraction of M437[L+M+] vs. control and M437[L+M-] vs. control analysis. The network was built utilizing the STRING database and a high confidence level of 0.8. The correlation strength is shown by the thickness of the line, the shape of node represents strains analyzed: square- M437[L+M+], diamond- M437[L+M-], circle means that protein was found in both strains. Color intensity changes are related to fold change, with blue representing downregulation and red representing upregulation.

Differentially expressed proteins, significantly regulated in L fractions of both strains, were incorporated in one protein-protein interaction network in the STRING database (Figure 5).

The network group consists of 56 nodes and 134 edges. Molecular complex detection (MCODE) clustering algorithm located four hubs of highly interconnected proteins. Functional analysis of these complexes related to the presence of LA virus revealed their participation in cytoplasmic translation, protein folding, proteasomal catabolic processes and protein trafficking. L fraction consists of 15 shared proteins between strains, 8 of which are found to be interconnected in created PPI network. Of the 26 unique proteins found in M437[L+M-], 16 proteins expanded obtained network. Group of proteins related to cytoplasmic translation (Rps22A, Rpp0, Rpp2B and others) and proteasomal catabolic process (Pre1,-9 and others) are positively up-regulated. Proteins involved in protein folding (Ssa1 and others) are down-regulated. The protein trafficking hub involves up-regulated Sec26, which is considered to play a critical role in processing and secretion of K1 toxin [27], Chc1, Syp1, and down-regulated proteins Sec21, Apl3, Spf1 altogether.

### 3.7. Cognate RNA Sequencing

**Figure 6:**
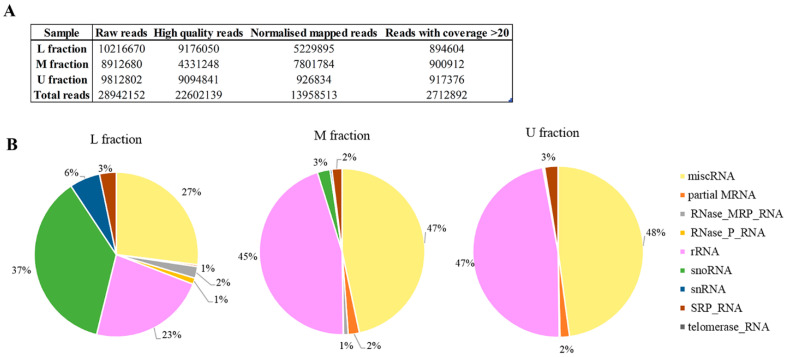
Total sequences obtained from [L+M-] strains samples and non-coding RNA composition. (**A**) Evaluation of ncRNA sequences in (U)-upper fraction without viral dsRNA, (M)- M2 viral dsRNA bearing fraction, (L)- LA ds RNA bearing fraction. (**B**) Pie chart representing the frequency of different non-coding RNA species in distinct fractions of [L+M-] strain.

Non-coding RNAs (ncRNAs) are important cellular regulators that modulate and control a wide range of physiological processes, including viral-host internal environment. RNA sequencing was used to display the virus-linked RNAs in investigated sample fractions of M437[L+M-] strain. We acquired a total of 28,942,152 raw reads; after filtering and mapping to yeast genome, 13,958,513 normalized reads were investigated (Figure 6A). The unmapped reads belong to LA-lus dsRNA. We utilized reference sequences with coverage greater than 20 for further investigation (Table S3). The most abundant RNA sequences (with 1000 and more reads) are accumulated across all M437[L+M-] sample fractions (Table 1). The highest number of reads belongs to NR_132216.1 and NR_132216.2 35S pre-ribosomal RNA, regardless of sample fraction investigated. The present study depicted the expression profiles of miscRNA, partial mRNA, the RNA subunit of the RNase mitochondrial RNA processing (MRP) enzyme complex (RNase_MRP_RNA), the RNA component of Ribonuclease P (RNase_P_RNA), rRNA, snoRNA, snRNA, the RNA component of the signal recognition particle (SRP_RNA), telomerase_RNA (Figure 6B). U and M fractions containing Gag protein without viral dsRNA are more similar in RNA composition than that of L fraction. In both U and M fractions, miscRNA (48% and 47%, respectively) and rRNA (47% and 45%, respectively) are dominant. In L fraction, miscRNA is 27%, rRNA 23%, and the dominating RNA category is snoRNA, which constitutes just 3% of the M fraction RNA, and less than 1% of the U fraction RNA. In addition, the L fraction contains 6% snRNA and 2% mitochondrial RNase MRP RNA, both of which are less than 1% in the other fractions. SRP_RNA is consistent between 2–3% in all fractions. Telomerase RNA was only identified in the M fraction.

## 4. Discussion

Proteomics studies of viruses is promising and productive research area. Virus needs to use genome replication and protein synthesis systems of its host to complete own life cycle. Following the principle of “guilty by association”, the analysis of direct interactions between the viral and host proteins holds promise for deciphering of the mechanisms behind virus survival. Here, insight on specific contacts is of imminent importance. Yeast viruses exhibit strong co-evolutionary relationships with their hosts and display no considerable effect on the host phenotype or growth traits, except for killer phenotype and transcriptional status [7,27,33,65,66]. Whole-cell proteomics studies often lack required resolution, making minor changes in proteome barely detectable. Therefore, we decided to proceed by preparing the virus capsid-enriched fractions of *Saccharomyces cerevisiae* M437- isogenic strains with different viral content.

The wild-type M437 strain possesses *Totiviridae* viruses LA-lus and M2, providing the strain with a killer phenotype [33]. To address the protein composition of each of these virus-bearing and virus-free yeast cell fractions of different *S. cerevisiae* strains, we eliminated either the M2 virus or both the LA-lus and M2 viruses [33] and performed proteomic analysis of each selected fraction by TMT labeling with following LC-MS/MS; note that deletion of LA-lus alone is not possible as it provides the replication machinery for the M2 virus. In such a way, we addressed the interactions of either virus in comparison to corresponding fractions of the virus-free strain. Isopycnic centrifugation, particularly involving CsCl gradient, is an established technique to prepare virus-related fractions [67,68]. The wealth of available data readily allows the distinction of viruses between yeast organelle–mitochondria in particular–content [69]. We took advantage of this method to target fractions, containing and lacking the genomic dsRNA and were also able to discern fractions with either LA-lus (4.6 kbp) or M2 (2.0 kbp) dsRNA (Figure 1). Although ultracentrifugation can separate LA-lus and M2 virions from cellular debris, contamination of the virion material by cellular components with comparable sedimentation characteristics cannot be ruled out. To exclude the unrelated proteins, we involved the samples prepared in parallel from a virus-free reference strain M437[L-M-]. The availability of three isogenic strains with various viral content ([L+M+], [L+M-] and [L-M-]) offers an unprecedented specificity and resolution to uncover the virus-host interactions with additional option of specific, virus type-related contacts. The exclusion of M virus from yeast killer strains is known to lead to the multiplication of LA virus copy number [12], thereby making an impact on the quantitative composition of proteins in fractions. Indeed, we observe this phenomenon by both SDS-PAGE (Figure 1) and proteomics analysis data of the M437[L+M-] strain (Figure 4).

In virus-containing fractions of the M437[L+M+] strain, we identified proteins belonging to ribosomes, proteasomes, protein folding and transport. In particular, the quantity of the ribosomal component of the 40S subunit Rps22Ap|Rps22Bp protein [70] increased along the increased amount of LA virus (Figure 4). In LA-1/M1 killer system, propagation of yeast viruses was found to depend on the concentration of free 60S ribosomal subunit and *MAK* genes [40]. Increase in Rpp0 and Rpp2B is detected in both M437 virus-bearing strains, however protein quantities slightly decrease when LA content increases. As discovered in yeast mutant strains lacking P1/P2 proteins–parts of ribosomal stalk, and parts of GTPase-associated-center which is directly responsible for stimulation of translation-factor-dependent GTP hydrolysis–significant propagation of the yeast L-A virus is observed. Additionally, the virus capsid proteins co-purify with ribosomal fraction using immunoaffinity chromatography [71]. The involvement of proteasomes in LA virus biology has never been observed before, though. The ribosomes and chaperone-related proteins are involved in biosynthesis of LA-lus Gag and GagPol proteins, while proteasomes control the turnover of the viral proteins, and transporting proteins regulate movement through endoplasmic reticulum and Golgi. These findings are in agreement with transcriptomics analysis data published previously [65], where virus-linked up-regulation was observed for ribosome part biosynthesis and RNA processing, while carbohydrate metabolism and ATP generation were found down-regulated. Reduction of 1,3-glucan receptor synthesis in M437[L+M+] strain is observed and might be attributed to the formation of resistance to killer toxin protein. It is of special importance the notice on the uniform regulation of proteins linked to M2 virus impact. While in M437[L+M-] strain, M fraction ribosomes are down-regulated (in M437[L+M+] up-regulated), proteasomes in M437[L+M+] M fraction are down-regulated and almost undetectable in M437[L+M-]. We attribute this to the result of killer toxin synthesis.

The protein-protein interaction network based on DEPs also observes the prominent granularity. 56 nodes and 134 edges resolve into four highly interconnected hubs. Functional analysis reveals participation in cytoplasmic translation, protein folding, proteasomal catabolic processes and protein trafficking, once again confirming the repertoire of principally targeted protein metabolism in a virus-bearing cell.

YEASTRACT analysis uncovered transcription factors behind the gene transcription of proteins present in our samples. The moderate number of identified TFs–ten in total–points on quite uniform regulation of transcriptomic landscape. Of note, nine of them were responsible for the activation of up-regulated proteins, whereas one is linked to the repression of down-regulated proteins. Among the most prominent activators, Cst6 controls expression of 62% of up-regulated proteins in M437[L+M+] strain and Met32 controls 88% of up-regulated proteins in M437[L+M-] cells. Cst6 is a transcriptional regulatory network member that responds to stress [72]. Met32 is involved in transcriptional regulation of methionine biosynthetic genes and is a potent cell cycle inhibitor during the stress response [73]. Whereas Yrr1-mediated repression is responsible for the majority of the down-regulated genes–48% in M437[L+M+] strain and 45% in M437[L+M-]. According to YEASTRACT database, there are a total of 1443 documented targets of Yrr1, an important regulator of multidrug resistance [74]. The precise mechanism(s) of action of identified TFs in the maintenance of *Totiviridae* viruses in yeast, if any, remains to be discovered.

By taking advantage of markedly increased amount of LA-lus virus inM437[LA+M-] strain, we decided to sequence total RNA content from all three fractions. Our initial intention was the discovery of tentative new “protoviruses”, given that RNA of increased molecular weight was observed to follow the CsCl density in a virion-containing fractions (not shown). However, the count numbers of individual sequences were below the threshold, indicating on significant variety of such sequences. Notably, LA-lus genome-missing samples U and M observe greater proportion of miscRNA (48% and 47%, respectively) than that for L fraction (27%). The latter fraction is enriched in snoRNA, making this type dominant and suggesting special, yet so far unresolved role in the LA-lus virus maintenance. The most dominating type of virion-associated RNAs is obviously 35S pre-ribosomal RNA and other, ribosome biogenesis-related RNAs. This observation appears in line with assortment of virus partners, observed by proteomics analysis–protein turnover components, ribosomal proteins including. Therefore, it is rather tempting to conclude the survival strategy for the ScV-LA virus as a close association with protein metabolism in the yeast cell, confirmed at both proteomics and RNA sequence analysis levels.

## 5. Conclusions

By performing high-resolution quantitative proteomics analysis on virus-linked subsets, we identified substantially enriched essential pathways of protein metabolism. The virus-linked host RNAs investigated by high-content sequencing led to identification of ribosomal RNAs inherently associated with LA-lus virus, along with other RNAs essential for ribosome biogenesis. This study provides a unique portrayal of yeast virions through the characterization of associated proteome and cognate RNAs, and offers a background for understanding ScV-LA viral infection persistency.

## Figures and Tables

**Table 1:** The most abundant RNA sequences with 1000 and more reads and coverage greater than 20.

L Fraction	M Fraction	U Fraction	Reference Sequence	Description
112788	208147	218849	NR_132216.1	S pre-ribosomal RNA (RDN37-2), miscRNA
112589	208148	218635	NR_132207.1	S pre-ribosomal RNA (RDN37-1), miscRNA
97599	2851	434	NR_132262.1	R17A (SNR17A), snoRNA
70020	1911	378	NR_132267.1	R17B (SNR17B), snoRNA
52351	1301	250	NR_132250.1	R19 (SNR19), snRNA
51789	78653	69740	NR_132213.1	S ribosomal RNA (RDN18-1), rRNA
51697	78390	69930	NR_132222.1	S ribosomal RNA (RDN18-2), rRNA
51035	125778	146671	NR_132218.1	S ribosomal RNA (RDN25-2), rRNA
51006	125576	146547	NR_132209.1	S ribosomal RNA (RDN25-1), rRNA
35044	2151	97	NR_132246.1	R11 (SNR11), snoRNA
30418	1890	234	NR_132159.1	R63 (SNR63), snoRNA
29439	17838	23351	NR_132171.1	R1 (SCR1), SRP_RNA
23026	1669	116	NR_132245.1	R83 (SNR83), snoRNA
20460	8160	610	NR_132251.1	E1 (NME1), RNase_MRP_RNA
18242	273	65	NR_132203.1	R42 (SNR42), snoRNA
16948	8176	434	NR_132194.1	R190 (SNR190), snoRNA
13009	234	36	NR_132254.1	R191 (SNR191), snoRNA
10818	528	319	NR_132195.1	R37 (SNR37), snoRNA
10796	708	876	NR_132166.1	R1 (RPR1), RNase_P_RNA
7633	1881	762	NR_132223.1	ternal transcribed spacer (ETS1-2), miscRNA
7604	1891	768	NR_132214.1	ternal transcribed spacer (ETS1-1), miscRNA
6566	611	242	NR_132204.1	R30 (SNR30), snoRNA
1425	526	300	NR_132154.1	R1 (LSR1), snRNA
1310	180	90	NR_132248.1	R86 (SNR86), snoRNA
1137	422	24	NR_132182.1	R46 (SNR46), snoRNA
816	2470	2839	NM_001184514.1	Tar1p (TAR1), partial mRNA

## Data Availability

Not applicable.

## Supplementary Materials

The following supporting information can be downloaded at: https://www.mdpi.com/article/10.3390/v14112345/s1, Figure S1: Principal component analysis (PCA) representing scores of different ultracentrifugation fractions of tree yeast strains and their biological replicates; Figure S2: Volcano plots displaying the proteins with differential expression discovered by contrasting virus-bearing vs. virus-free cell fractions; Table S1: List of the differentially expressed proteins (DEPs) detected in fractions; Table S2: Biological process (BP), Molecular function(MF) and KEGG enrichment analysis of statistically significantly up- and down-regulated DEPs; Table S3: lncRNA sequences of different samples mapped to *Saccharomyces cerevisiae* RNA transcriptome.
